# Parliamentary workload and outpatient medical costs among Japanese national civil servants: An ecological study in Kasumigaseki

**DOI:** 10.5271/sjweh.4338

**Published:** 2026-11-01

**Authors:** Koryu Sato, Hirotaka Kato, Kosuke Inoue, Naoki Kondo, Ichiro Kawachi

**Affiliations:** 1 Faculty of Policy Management, Keio University, Kanagawa, Japan.; 2 School of Economics and Business Administration, Yokohama City University, Kanagawa, Japan.; 3 Department of Health Promotion and Behavioral Sciences, Graduate School of Medicine and School of Public Health, Kyoto University, Kyoto, Japan.; 4 Department of Social Epidemiology, Graduate School of Medicine and School of Public Health, Kyoto University, Kyoto, Japan; 5 Department of Social and Behavioral Sciences, Harvard T.H. Chan School of Public Health, Boston MA, United States.

**Keywords:** administrative claim, healthcare utilization, long working hours, Japan

## Abstract

**Objectives:**

This study aimed to examine whether ministry-level parliamentary workload was associated with outpatient medical costs among Japanese national civil servants, who are exempt from the Labor Standards Act’s statutory limits on working hours.

**Methods:**

We conducted an ecological study of headquarters branches (“Kasumigaseki”) of ten major cabinet-level ministries, 2016–2023. Participants were Japanese national civil servants aged 18–50 years. The analysis included 6454 outpatient claims adjudicated in April of each year. The outcome was sex- and age-standardized per-capita monthly outpatient costs, estimated using Horvitz–Thompson weighting. Inter-ministry differences were decomposed by ICD-10 category. Parliamentary (*Diet*) workload was annual deliberation time of standing *Diet* committees responsible for each ministry’s policy areas, divided by insured headquarters members. Weighted least-squares regression examined the association between prior-year deliberation time and standardized outpatient costs.

**Results:**

Standardized per-capita outpatient costs varied more than twofold across ministries, from $19.8/month at the Ministry of Foreign Affairs to $42.7/month at the Ministry of Health, Labour and Welfare (MHLW). At MHLW, mental and behavioral disorders accounted for the largest above-average cost component. MHLW also had the longest deliberation time. Adjusting for year fixed effects, a 1% increase in deliberation time per civil servant was associated with a 0.25% increase in standardized per-capita outpatient costs (95% CI 0.04–0.46), driven by claims per capita rather than cost per claim.

**Conclusions:**

Greater parliamentary workload was associated with higher outpatient medical costs among Japanese national civil servants. Enhanced occupational health monitoring and support in ministries with persistently high workloads may be worth considering.

In 2016, 488 million people were burdened by long working hours in the world (1). The health consequences of long working hours are well documented. Large-scale systematic reviews and meta-analyses have demonstrated robust associations between long working hours and the risk of cardiovascular diseases (2–4). Among Asian populations, long working hours are also associated with incident depression (5). In most developed countries, labor legislation has been strengthened over the decades to regulate working hours and protect employees from excessive workloads.

Japan presents a particularly intriguing case. Marmot and Davey Smith once suggested that Japan’s distinctive work organization, including workers’ commitment to the workplace and employers’ commitment to workers, might have contributed to the country’s favorable health profile (6). The same organizational commitment, however, may have a dark side. Japan’s national civil servants are exempt from the Labor Standards Act and therefore fall outside its statutory protections, leaving them exposed to persistently long working hours. A former civil servant – who worked in the Ministry of Health, Labour and Welfare (MHLW) which oversees the Labor Standards Act – recounted working 150–200 overtime hours per month (7). Even on Christmas Eve, the lights in the building housing the MHLW burn bright (supplementary figure S1). Responding to questions raised during parliamentary debate represents one of the most burdensome aspects of Japanese national civil servants’ work. Questions from Japan's Parliament (the *Diet*) members are often notified the evening before a committee session. As a result, ministries must identify responsible divisions, prepare draft answers, clear them through senior officials, and brief ministers overnight. A Cabinet Bureau of Personnel Affairs survey documented that, during a 2022 *Diet* session, the average time at which ministries could begin drafting the final set of answers was 19:08 hours on the previous working day, and all answers were completed only at 05:06 hours the following morning (8). A study using mobile phone location data from the Kasumigaseki district (the Japanese equivalent of Whitehall) found that responding to a single additional written parliamentary question results in more than 30 civil servants remaining in the office past 23:00 hours, with the effect persisting well beyond the statutory one-week response deadline (9). According to Karasek’s Job Demand–Control model, occupations characterized by high demands and low control are associated with the highest levels of stress (10). Applying this framework, Japanese national civil servants are likely to be exposed to a particularly stressful occupational environment: they must respond promptly and accurately to enquiries from legislators, while individual officials have limited discretion over either the timing or substance of these demands.

Yet little research has examined the health consequences of over-work among Japanese national civil servants. Although individual-level clinical outcomes such as incident depression, cardiovascular disease, biomarkers, or disease control would be desirable, there is no equivalent of a Whitehall Study in Japan to prospectively study the association between working conditions and health outcomes among civil servants. However, in Japan’s universal health insurance system, outpatient care is universally covered and reimbursed according to a nationally uniform fee schedule, making claims-based outpatient costs a useful, though imperfect, indicator of healthcare utilization and possible excess health burden. These costs do not directly measure morbidity, but they provide one of the few observable outcomes available for comparing ministries.

Using unique claims data, this study describes outpatient medical costs as an indicator of healthcare utilization and possible health burden among Japanese national civil servants working in Kasumigaseki. Additionally, we investigate the ministry-level association of outpatient costs with parliamentary deliberation time in the relevant standing *Diet* committees as a proxy of job demands within each ministry.

## Methods

### Claims data

Japan has universal health insurance coverage. All full-time national civil servants are mandatorily enrolled in the Mutual Aid Associations for National Public Officials, the employer-based insurers for the national government workforce; there are therefore no uninsured full-time employees in the ministries studied. A claim is generated whenever an insured member receives any insured medical service. Japan’s health system has no gatekeeping, and patients have direct access to both clinics and hospitals. Hence, claims capture all reimbursed physician visits including primary and specialist care. The prices of all insured services are set by a nationwide uniform fee schedule, and the net change at each biennial revision during the study period was <1%.

Claims data were obtained from the *Survey on Medical Care Utilization in Mutual Aid Associations of the Federation*, conducted annually by the Ministry of Finance (MOF). Each survey year, approximately 5% of claims adjudicated in April (89.0% of claims pertain to care received in February) are randomly sampled from all Mutual Aid Associations for National Public Officials. Each claim record contains the member’s sex, birth year and month, diagnosis codes, and medical fees, but no personal identifiers. MOF provided data covering April 2016 through April 2023 for the present study.

We analyzed claims from the following ten ministries with a large number of insured members: the Cabinet Office (CAO), the Ministry of Internal Affairs and Communications (MIC), the Ministry of Justice (MOJ), the Ministry of Foreign Affairs of Japan (MOFA), the MOF, the Ministry of Education, Culture, Sports, Science and Technology (MEXT), the MHLW, the Ministry of Agriculture, Forestry and Fisheries (MAFF), the Ministry of Economy, Trade and Industry (METI), and the Ministry of Land, Infrastructure and Transport (MLIT). The Ministry of Defense was excluded from the analysis, as its insured members include many Self-Defense Forces personnel whose working conditions differ substantially from those of other ministries. To focus on civil servants directly exposed to parliamentary workload, we restricted analysis to claims from the headquarters branch of each ministry located in Kasumigaseki. While civil servants aged 18–50 years were included, those aged >50 were excluded because they are frequently seconded to regional or affiliated agencies where parliamentary pressure is lower (11). Because only 57 inpatient claims were identified over the eight-year period, analysis was limited to outpatient claims. After applying these criteria, 6454 outpatient claims from ten ministries were available for analysis.

As the data were anonymized and did not contain personally identifiable information, individual consent and institutional ethical review for their secondary use were not required under Japanese law.

### Per-capita outpatient medical cost

The numerator of per-capita outpatient cost was computed as a Horvitz–Thompson estimate; each claim’s medical fee was divided by the annual, ministry-specific sampling rate. To limit the influence of extreme values, weighted costs were winsorized at the 99th percentile. Japanese yen were converted to US dollars at an approximate average exchange rate of ¥120 per USD over the study period (2016–2023). The denominator was the total number of insured headquarters branch members as of February of each year, obtained from the *Annual Report of Mutual Aid Associations for National Public Officials* by ministry and survey year. The data was provided by MOF for the present study.

Because sex- and age-specific membership counts for the headquarters branch are not available, we estimated them by applying the annual sex and age distribution of all branches combined to the total headquarters branch membership for each association. This procedure assumes that the sex and age distribution of headquarters branch members was similar to that of all branches within the same ministry.

In the Mutual Aid Associations of MAFF and METI, the headquarters branch and the local branches were merged into a single administrative unit within each association from fiscal year 2020 onward. For these two ministries, we estimated post-2020 headquarters membership by applying the pre-2020 headquarters-to-total membership ratio to total membership in each subsequent year. Claims costs were adjusted using the same ratio. As this procedure may introduce measurement error, we conducted a sensitivity analysis excluding MAFF and METI.

### Per-capita parliamentary deliberation time

The National *Diet* has standing committees in both the House of Representatives and the House of Councillors, each of which deliberates on matters under the jurisdiction of the relevant ministries (12, 13). We defined parliamentary deliberation time as the total time that these standing committees spent in formal sessions during each calendar year. Deliberation time for each standing committee from January 2015 to December 2022 was retrieved by web-scraping the National *Diet* Record Search System (14). Each standing committee was mapped to the relevant ministry based on its mandate (supplementary table S1). We did not include deliberation time in the Budget Committees, because these committees cover government-wide budgetary matters involving all ministries, making it difficult to attribute their deliberation time to any specific ministry. Per-capita deliberation time was calculated by dividing the total annual deliberation time by the number of insured headquarters members, consistent with the denominator used for per-capita outpatient costs.

We used annual committee deliberation time as a ministry-level proxy for job demands. This seems a reasonable assumption because deliberation time tends to be longer when a ministry has many bills and pending issues to handle. In the Japanese central government, Diet committee sessions require ministries to prepare answers, briefing materials, background documents, and ministerial briefings, often under short deadlines. Questioning time is generally allocated to individual legislators in short blocks, commonly around 15–30 minutes (14). Longer committee deliberation time therefore generally implies that more legislators had opportunities to question ministers and government officials, and is likely to be correlated with the number and complexity of questions that ministries must prepare for and respond to. In addition, delayed question notification imposes workload on a wider set of officials than those ultimately responsible for drafting the final answers, because potentially relevant divisions may need to remain available until the questions are received, allocated, and narrowed to the responsible units.

### Statistical analysis

Sex- and age-standardized per-capita costs for each ministry were calculated by the direct method, using the pooled population across all ten ministries and all eight survey years as the standard. Age was categorized into two groups to reduce the influence of high-cost claims within sparse age-specific strata (18–40 and 41–50 years). For each sex–age–ministry–year stratum, per-capita costs were multiplied by the stratum’s standard weight and summed to produce the ministry-specific standardized estimate. Uncertainty in the standardized estimates was quantified by a parametric bootstrap with 2000 iterations. The same standardization procedure, applied within each ministry-year, provided the outcome for the regression analysis described below.

To understand which disease categories drove inter-ministry cost differences, standardized per-capita costs were calculated separately for each of the ICD-10 chapters. Each ministry’s chapter-specific standardized cost was subtracted from the corresponding cross-ministry mean for that chapter, yielding a contribution score. Contributions summing to positive values indicate above-average cost in that disease category.

Our primary estimand was the ministry-level ecological association between prior-year parliamentary deliberation time per insured headquarters member and standardized per-capita outpatient medical cost in the following year. The analysis did not estimate the individual-level effect of working hours on health, because individual working hours and direct involvement in parliamentary duties were unavailable. The unit of regression analysis was the ministry-year (ten ministries observed over eight years). We fitted weighted least-squares regression models, weighting each ministry-year by the inverse of the estimated variance of its standardized cost on the log scale, approximated by the delta method. This approach assigned greater influence to ministry-years with more precise cost estimates.

The primary outcome was log-transformed standardized per-capita outpatient costs, and the exposure was log-transformed total annual deliberation time per insured member in the previous year. Regression coefficients therefore represent the percentage change in per-capita cost associated with a 1% increase in deliberation time per member. In model 1, sex- and age-standardized per-capita costs were regressed on deliberation time; model 2 adjusted year fixed effects that absorb all time trends across ministries, such as revisions of the medical fee schedule and changes in demands of care due to the COVID-19 pandemic; model 3 additionally adjusted for ministry fixed effects that absorb all time-invariant ministry characteristics, such as policy domain, organizational culture, and workforce selection at recruitment. Per-capita cost can be decomposed into claims per capita and cost per claim, and we also regressed them on deliberation time in each model. Standard errors and 95% confidence intervals (CI) were estimated using CR2 cluster-robust variance estimation with the Satterthwaite degrees of freedom, clustered at the ministry level (15, 16). All analyses were conducted in R version 4.6.0 (R Foundation for Statistical Computing, Vienna, Austria).

### Sensitivity analysis

To check robustness of the association, we performed several sensitivity analyses based on the model adjusted for year fixed effects. First, we re-estimated the model after excluding each ministry in turn to see which ministry influenced the association. Second, we included civil servants aged 51–64 years in the analysis, who are less likely to be affected by parliamentary pressure. Third, we used claims data without winsorization and with winsorization at the 97.5^th^ and 95^th^ percentiles. Fourth, we replaced the one-year lag with a two-year lag, modelling outpatient costs in a given year against deliberation time two years earlier, and extended the committee deliberation series back to 2014 so that the full outcome period was retained.

## Results

As shown in table 1, 252 830 insured member-years were included across the ten ministries (68.6% male; 43.9% aged 41–50 years). Cumulative parliamentary deliberation time over the eight-year study period was longest for MHLW (2534 hours), substantially exceeding the second-highest ministry (CAO: 1528 hours). In terms of deliberation time per 100 civil servants, MOJ had the longest at 14.67 hours, followed by MHLW at 9.16 hours, while MOFA had the shortest at 2.38 hours. The mean of claims per capita (claims rate) was 0.44 claims per person per month, ranging from 0.26 (MOFA) to 0.59 (MOJ). Mean cost per claim was $73.2, ranging from $67.4 (MOF) to $77.3 (MOFA).

**Table 1:** Descriptive statistics by ministry. [CAO=Cabinet Office; MIC=Ministry of Internal Affairs and Communications; MOJ=Ministry of Justice; MOFA=Ministry of Foreign Affairs of Japan; MOF=Ministry of Finance; MEXT=Ministry of Education, Culture, Sports, Science and Technology; MHLW=Ministry of Health, Labour and Welfare; MAFF=Ministry of Agriculture, Forestry and Fisheries; METI=Ministry of Economy, Trade and Industry; MLIT=Ministry of Land, Infrastructure and Transport].

Ministry	Insured member		Men		Age 41–50 years		Deliberation time		Deliberation time per 100 members		Number of claims		Claims per capita		Cost per claim		Cost per capita
	N		%		%		Hours		Hours		N		N		USD		USD
CAO	25 334		64.5		40.0		1528		6.03		626		0.50		72.1		36.1
MIC	27 377		74.0		40.9		997		3.64		564		0.41		71.0		29.0
MOJ	9212		69.8		43.6		1352		14.67		268		0.59		71.8		42.0
MOFA	39 459		63.1		41.7		938		2.38		502		0.26		77.3		20.2
MOF	12 481		78.6		42.0		1031		8.26		224		0.36		67.4		24.6
MEXT	16 129		59.6		39.6		921		5.71		392		0.51		73.8		37.5
MHLW	27 663		67.2		48.6		2534		9.16		786		0.57		73.4		42.1
MAFF	21 449		68.0		55.3		1109		5.17		1 330		0.54		75.3		40.7
METI	39 103		66.6		44.5		1038		2.66		984		0.38		71.0		27.1
MLIT	34 624		77.4		42.8		1056		3.05		778		0.46		74.8		34.6
Total	252 830		68.6		43.9		12 503		4.95		6 454		0.44		73.2		32.0

Figure 1 shows sex- and age-standardized per-capita outpatient costs by ministry with 95% bootstrap CI. MHLW had the highest standardized cost ($42.7/month), followed by MOJ ($42.1/month) and MAFF ($38.6/month). MOFA had the lowest cost ($19.8/month), followed by MOF ($25.1/month) and METI ($27.0/month). Annual per-capita outpatient costs by ministry are presented in supplementary figure S2. Figure 2 presents the contribution of each ICD-10 disease chapter to the ministry-level deviation from the cross-ministry mean. For MHLW, the dominant contributor to above-average costs was mental and behavioral disorders. MOJ, which had the second-highest per-capita cost, showed elevated contributions from neoplasms, respiratory diseases, and digestive system diseases.

**Figure 1:**
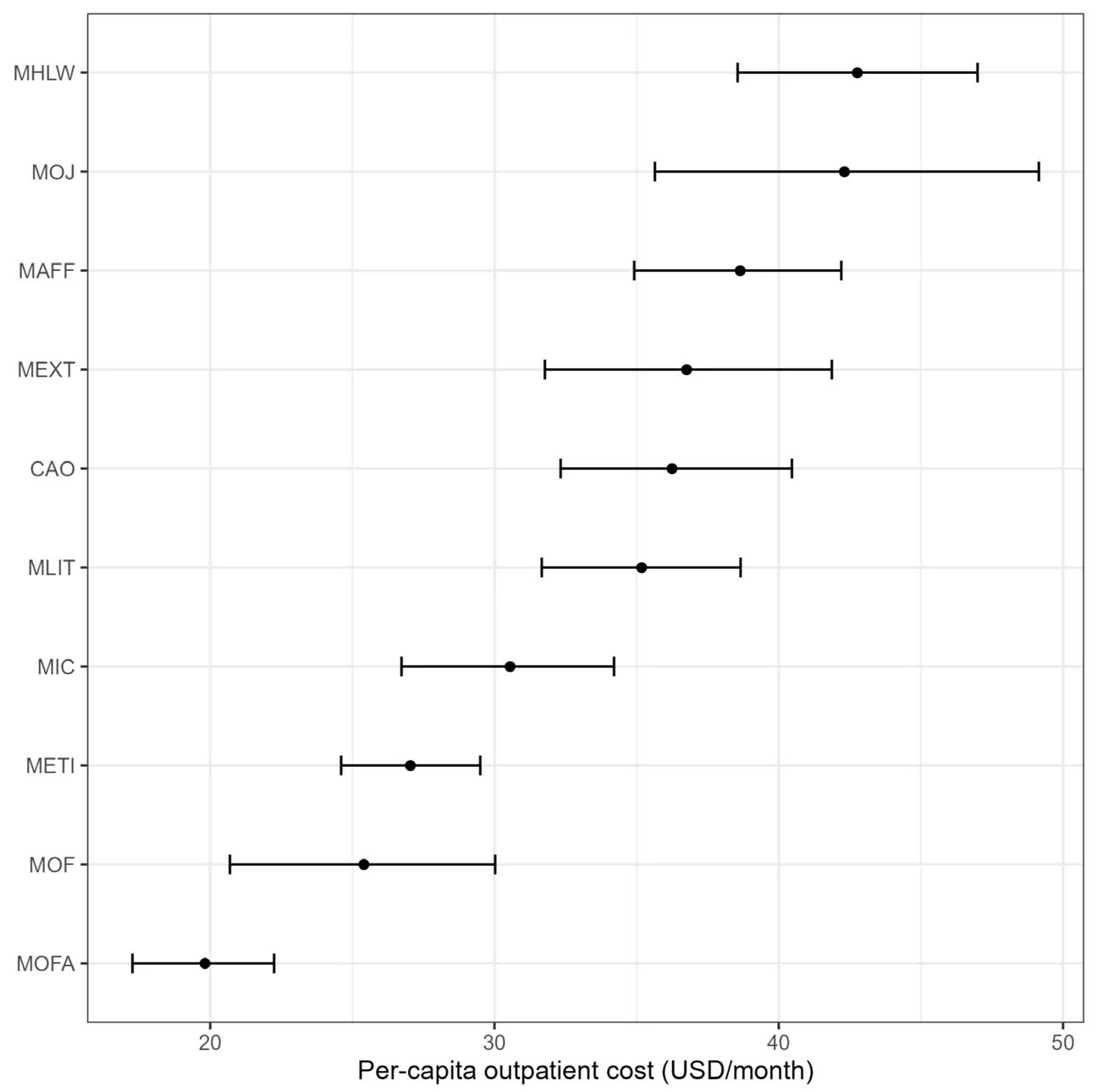
Per-capita outpatient costs by ministry (2016–2023). Per-capita outpatient costs are standardized by sex and age groups. Horizontal bars indicate 95% bootstrap confidence intervals. [CAO=Cabinet Office; MIC=Ministry of Internal Affairs and Communications; MOJ=Ministry of Justice; MOFA=Ministry of Foreign Affairs of Japan; MOF=Ministry of Finance; MEXT=Ministry of Education, Culture, Sports, Science and Technology; MHLW=Ministry of Health, Labour and Welfare; MAFF=Ministry of Agriculture, Forestry and Fisheries; METI=Ministry of Economy, Trade and Industry; MLIT=Ministry of Land, Infrastructure and Transport].

**Figure 2:**
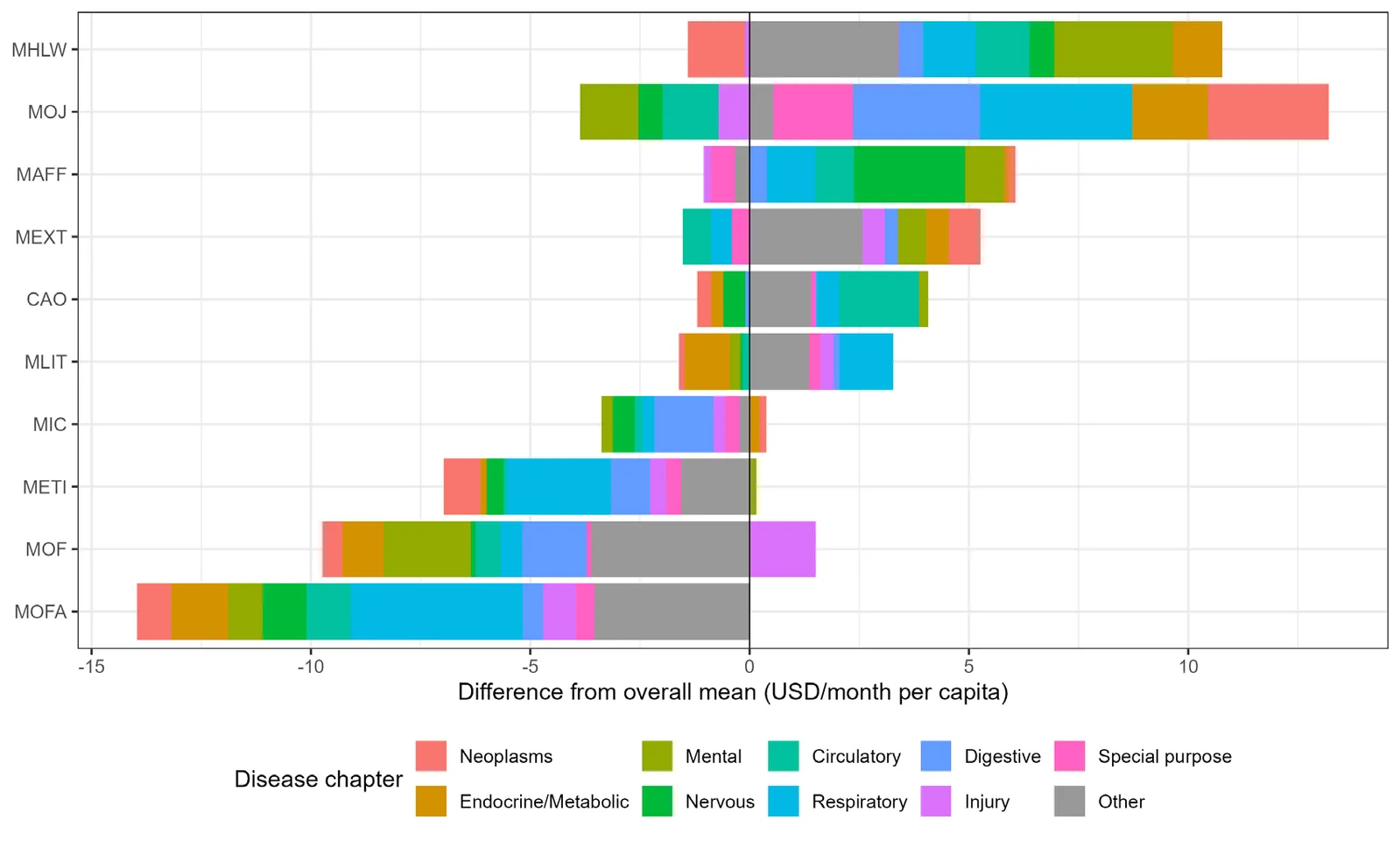
Contribution of ICD-10 disease chapters to ministry-level cost differences. Bars represent each ministry’s standardized per-capita cost in a given disease chapter minus the cross-ministry mean for that chapter; positive values indicate above-average expenditure. [CAO=Cabinet Office; MIC=Ministry of Internal Affairs and Communications; MOJ=Ministry of Justice; MOFA=Ministry of Foreign Affairs of Japan; MOF=Ministry of Finance; MEXT=Ministry of Education, Culture, Sports, Science and Technology; MHLW=Ministry of Health, Labour and Welfare; MAFF=Ministry of Agriculture, Forestry and Fisheries; METI=Ministry of Economy, Trade and Industry; MLIT=Ministry of Land, Infrastructure and Transport].

Figure 3 illustrates a scatter plot showing the relationship between parliamentary deliberation time and standardized outpatient costs. Each point represents data for a given ministry in a given year. Table 2 presents the association between them. In the model adjusting for year fixed effects (model 2), a 1% increase in deliberation time per member was associated with a 0.25% increase in standardized per-capita outpatient costs (95% CI 0.04–0.46). When cost per capita was decomposed, claims per capita were associated with deliberation time (0.26, 95% CI 0.02–0.50), whereas cost per claim was not (-0.004, 95% CI -0.07–0.06). In the model additionally adjusting for ministry fixed effects (model 3), no clear associations were observed. This loss of precision was expected because between-ministry differences accounted for 83.5% of the total variance in deliberation time, leaving little within-ministry variation to identify the fixed-effects estimate. The association observed in model 2 should therefore be understood as reflecting primarily persistent between-ministry differences. Within-ministry trajectories of deliberation time and standardized outpatient costs, and the corresponding deviations from ministry-specific means, are shown in supplementary figures S3 and S4; consistent with model 3, the within-ministry association was weak.

**Table 2:** Association between deliberation time and standardized outpatient costs. [CI=confidence intervals using CR2 ministry-cluster-robust variance estimation with Satterthwaite degrees of freedom].

	Model 1 ^a^		Model 2 ^b^		Model 3 ^c^
	Estimate (95% CI)		Estimate (95% CI)		Estimate (95% CI)
Cost per capita	0.23 (0.03–0.44)		0.25 (0.04–0.46)		0.07 (-0.30–0.44)
Claims per capita	0.26 (0.04–0.48)		0.26 (0.02–0.50)		-0.06 (-0.38–0.25)
Cost per claim	-0.02 (-0.09–0.04)		-0.004 (-0.07–0.06)		0.14 (-0.07–0.34)

^a^ Unadjusted model includes deliberation time alone. ^b^ Adjusted for year fixed effects (FE). ^c^ Adjusted for year and ministry FE.

**Figure 3:**
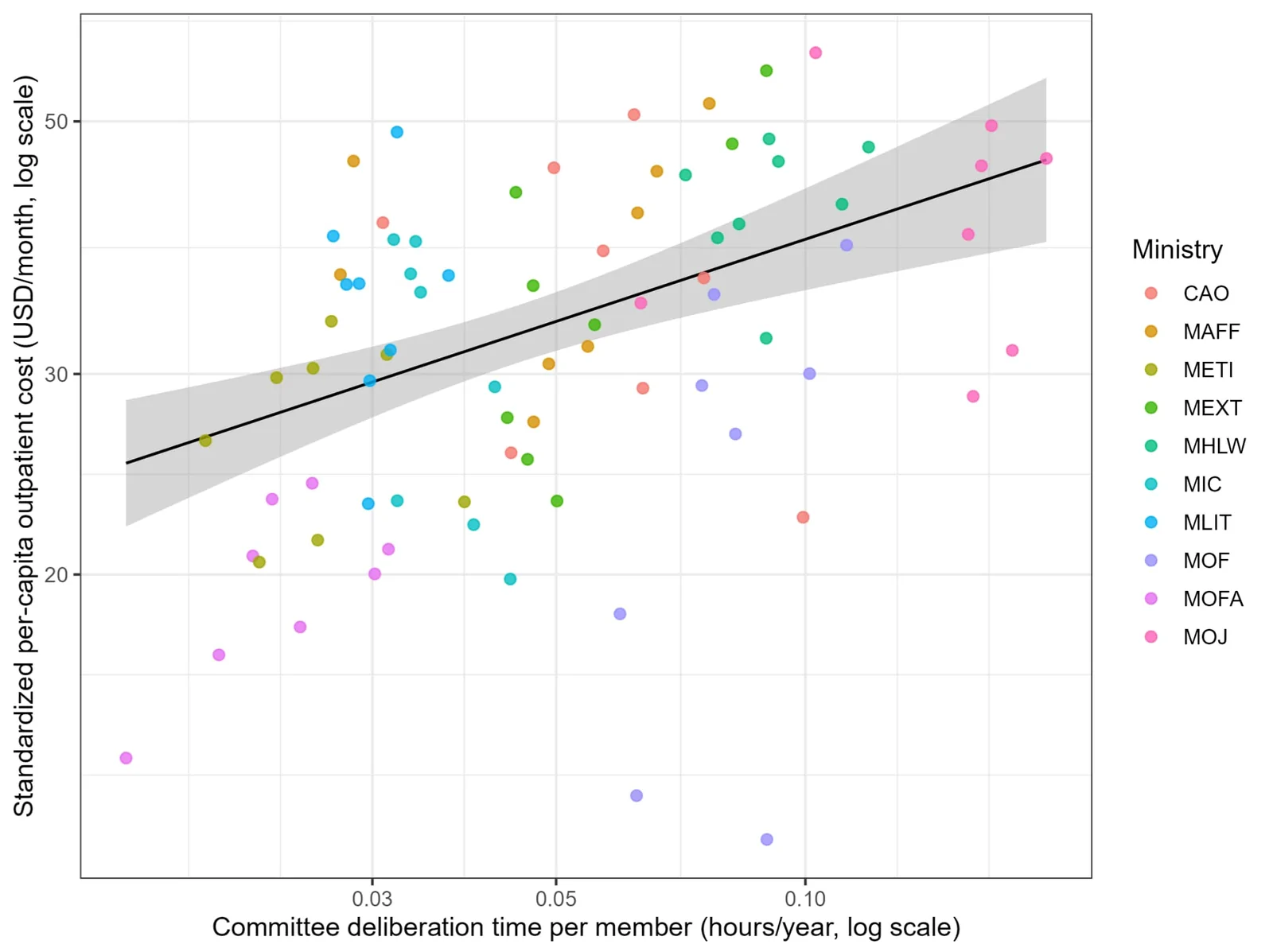
Outpatient costs by deliberation time. Each point represents one ministry-year observation. Both axes are shown on a log scale. The solid line indicates the fitted linear regression line, and the shaded area indicates the 95% confidence interval. [CAO=Cabinet Office; MIC=Ministry of Internal Affairs and Communications; MOJ=Ministry of Justice; MOFA=Ministry of Foreign Affairs of Japan; MOF=Ministry of Finance; MEXT=Ministry of Education, Culture, Sports, Science and Technology; MHLW=Ministry of Health, Labour and Welfare; MAFF=Ministry of Agriculture, Forestry and Fisheries; METI=Ministry of Economy, Trade and Industry; MLIT=Ministry of Land, Infrastructure and Transport].

Supplementary figure S5 summarizes the estimates in sensitivity analyses. Excluding ministries with the highest and lowest outpatient costs attenuated the associations; the estimated coefficient was 0.21 (95% CI -0.01–0.44) when MHLW was excluded, while it was 0.18 (95% CI -0.03–0.39) when MOFA was excluded. Including civil servants aged 51–64 years also attenuated the association (0.06, 95% CI -0.10–0.22) as expected. The other sensitivity analyses produced similar results to the main analysis (model 2).

## Discussion

To our knowledge, this study is the first to describe ministry-level variation in outpatient medical costs among Japanese national civil servants and to examine its association with parliamentary deliberation workload. We found that ministries with longer deliberation time per member tended to have higher subsequent outpatient costs. The association was driven by claims per capita rather than cost per claim.

The substantial inter-ministry heterogeneity in outpatient costs indicates that healthcare utilization among national civil servants is not uniform. Even after standardization for sex and age, costs varied more than twofold across ministries ($19.8–42.7/month), suggesting that the variation is not explained by demographic composition alone and may partly reflect occupational exposures. MHLW illustrates this pattern most clearly, with both the highest per-capita outpatient costs and the longest parliamentary deliberation time. The cumulative deliberation time of its overseeing committee exceeded that of the second-busiest committee by >1000 hours over the study period, indicating that the workload gap between MHLW and other ministries is substantial. The elevated costs at MHLW were driven particularly by mental health-related costs, congruent with the literature linking long working hours to mental health (5, 17). At the same time, the MHLW result warrants caution. The study period encompassed the COVID-19 pandemic and the irregularities in official government statistics, both of which involved MHLW and plausibly increased not only parliamentary scrutiny but also other forms of work. Under our interpretation of deliberation time as a marker of overall ministry-level workload, however, these events form part of the workload that the marker is intended to capture.

The irony is difficult to miss: the ministry responsible for protecting workers from overwork appeared, in our ecological analysis, to sit at the top of the healthcare-utilization table. This irony has also been visible outside our data. A national media report described a pregnant MHLW official working past 03:00 hours while on standby for *Diet*-related work and legislative duties (18). The anecdote is not causal evidence, but it captures the paradox at the center of this study: the institution expected to lead Japan’s work-style reform may itself be exposed to unusually intense, externally imposed workload. This matters not only for civil servants. When the ministry responsible for labor standards struggles to embody those standards, the credibility and normative force of work-hour regulation may be weakened across the wider labor market.

MOJ had the second-highest costs. In terms of deliberation time per member, MOJ ranked highest among the ten ministries, consistent with the possibility that parliamentary burden may contribute to higher healthcare utilization in this ministry. The decomposition analysis further showed that MOJ’s above-average costs were driven not by a single disease category but by elevated contributions from multiple categories, such as neoplasms, respiratory disease, and digestive system disease. This pattern suggests that the high outpatient costs at MOJ may reflect a broad burden of physical health conditions, rather than a ministry-specific increase in mental health-related costs alone.

MOFA had both the shortest deliberation time per member and the lowest per-capita outpatient cost, again suggesting a positive relationship between parliamentary deliberation burden and outpatient costs. By contrast, MOF had the third-longest deliberation time per member but the second-lowest per-capita outpatient cost. Given the low claims rate among MOF staff, we cannot rule out the possibility that heavy workload limited opportunities to seek medical care. However, deliberation time per member was positively correlated with the claims rate in our analysis, suggesting that, at the ministry level, longer deliberation time did not appear to be associated with lower healthcare-seeking. METI had the third-lowest per-capita cost. Notably, in 2026, METI became the first government agency to be certified as a Health & Productivity Management Organization (a national recognition program for employers who strategically invest in employee health) (19). Although this certification postdates our study period and cannot explain the observed estimates directly, it illustrates that ministry-level occupational health practices may differ.

Several limitations should be acknowledged. First, the survey captures approximately 5% of claims adjudicated in April each year. Owing to this limitation, the number of inpatient claims was insufficient for analysis, and we were therefore restricted to examining outpatient claims alone. Furthermore, because the majority of the claims pertained to care received in February, seasonal variation could not be accounted for. Second, causal inference from our ecological design is inherently limited. The Job Demands–Resources model holds that the effect of job demands depends on the resources available to workers, including support from supervisors and colleagues, team climate, and role clarity (20). We could not consider such potential confounders. Additionally, claims-based outpatient costs may not be a perfect proxy for health status, as they reflect not only morbidity but also healthcare-seeking behavior and access to care. Thus, higher outpatient costs may reflect not only poorer health but also greater awareness of symptoms or easier access to care, whereas lower costs may partly reflect unmet healthcare needs. The observed association should therefore be interpreted as a ministry-level association rather than as definitive evidence of an individual-level causal effect. Third, the headquarters branch classification is imperfect; some staff nominally registered at headquarters may be seconded to regional agencies where parliamentary workload is lower. This tendency is considered to be particularly strong among civil servants aged >50 years, and we therefore excluded them from the main analysis. This misclassification would tend to attenuate estimated associations. Fourth, because the claims are anonymized cross-sections, we could not determine whether multiple claims originated from the same individual, nor adjust for members’ prior health status or previous healthcare utilization. Ministries may therefore differ in the prevalence of chronic conditions that generate repeated outpatient visits among a small number of members. Fifth, we could not distinguish between types of parliamentary workload. According to the challenge–hindrance stressor framework, challenge stressors can enhance employee motivation and performance, whereas hindrance stressors undermine both (21, 22). For example, deliberation on legislation may constitute a challenge stressor, whereas responding to crises or administrative scandals may constitute a hindrance stressor. Although both types of stressor generate comparable levels of strain (22), they differ in their motivational consequences, and the long-term health consequences may therefore depend on the content of parliamentary deliberation. Despite these limitations, this study makes a unique contribution by shedding light on healthcare utilization and possible health burdens among Japanese national civil servants, an issue that has hitherto remained largely unexamined.

In summary, this study demonstrates substantial variation in per-capita outpatient medical costs across Japanese central government ministries and finds a positive association between parliamentary deliberation time and outpatient costs. The findings suggest that parliamentary workload may serve as a marker of occupational health burden among civil servants, with potential implications for healthcare utilization. Further studies using individual-level data on working hours, job stress, and health outcomes are needed to clarify the occupational health implications of parliamentary workload. In the meantime, monitoring healthcare needs and strengthening occupational health support in ministries with persistently high workloads may be worth considering.

## Supplementary material

Supplementary material 1 Supplementary material
