# Supplementary material for: Parliamentary workload and outpatient medical costs among Japanese national civil servants: An ecological study in Kasumigaseki
Source: Scand J Work Environ Health. 2026 Oct 26;52(6):680–8. doi: 10.5271/sjweh.4338 (PMC13733483; doi:10.5271/sjweh.4338)
Supplement: Supplementary file 1 [file SJWEH-52-6-680-s001.pdf]

Parliamentary workload and outpatient medical costs among Japanese national civil servants: An ecological study in Kasumigaseki

by Koryu Sato, Hirotaka Kato, Kosuke Inoue, Naoki Kondo, Ichiro Kawachi

Supplementary Materials

Figure S1. Central Government Building No. 5, housing the Ministry of Health, Labour and Welfare. 7:00 p.m., December 24, 2025

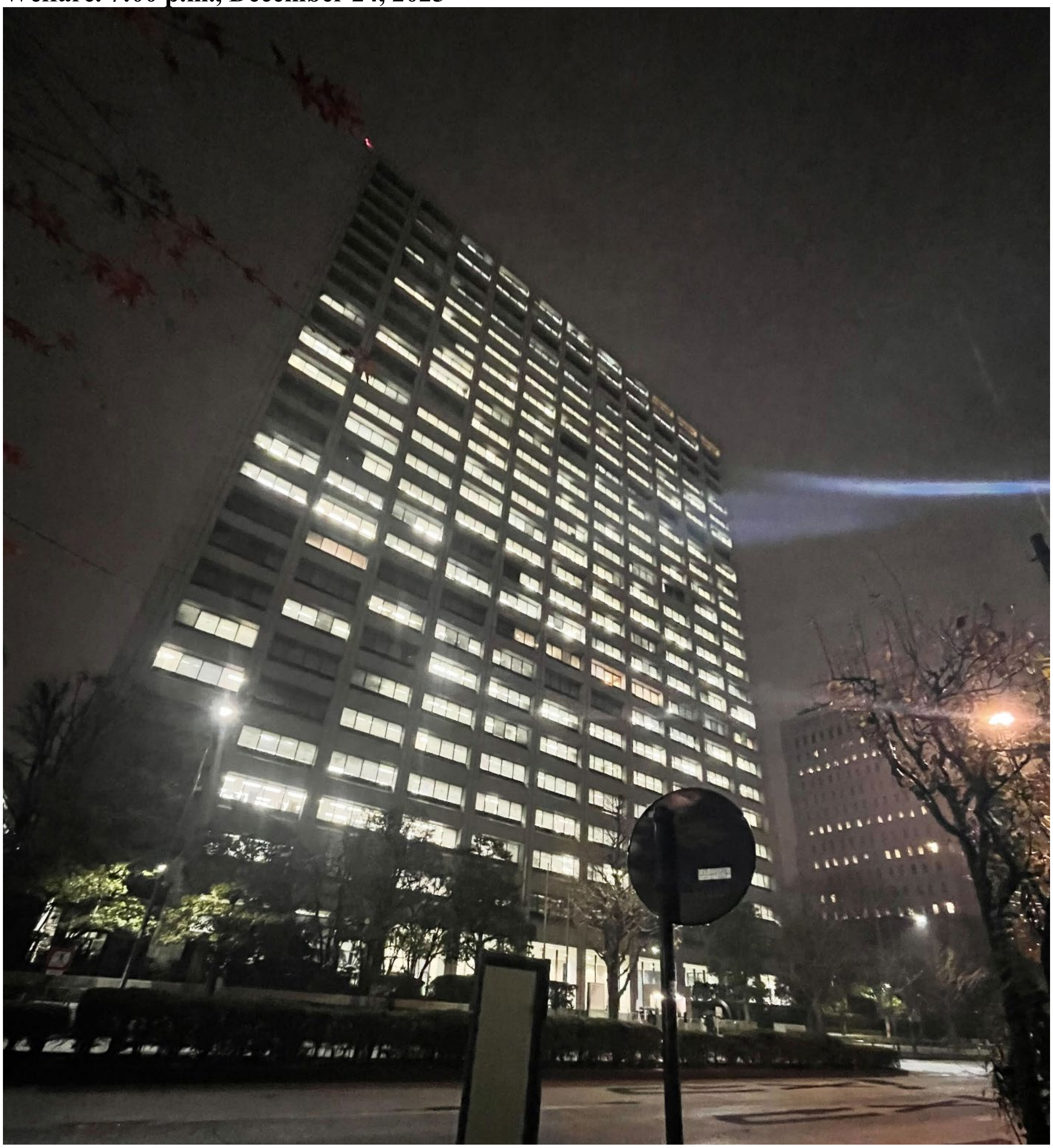

Table S1. Mapping between standing Diet committees and ministries

Chamber	Standing Diet committee	Mapped ministry
House of Representatives	Committee on Cabinet	CAO
House of Councillors	Committee on Cabinet	CAO
House of Representatives	Committee on Internal Affairs and Communications	MIC
House of Councillors	Committee on Internal Affairs and Communications	MIC
House of Representatives	Committee on Judicial Affairs	MOJ
House of Councillors	Committee on Judicial Affairs	MOJ
House of Representatives	Committee on Foreign Affairs	MOFA
House of Councillors	Committee on Foreign Affairs and Defense	MOFA
House of Representatives	Committee on Financial Affairs	MOF
House of Councillors	Committee on Financial Affairs	MOF
House of Representatives	Committee on Education, Culture, Sports, Science and Technology	MEXT
House of Councillors	Committee on Education, Culture and Science	MEXT
House of Representatives	Committee on Health, Labour and Welfare	MHLW
House of Councillors	Committee on Health, Labour and Welfare	MHLW
House of Representatives	Committee on Agriculture, Forestry and Fisheries	MAFF
House of Councillors	Committee on Agriculture, Forestry and Fisheries	MAFF
House of Representatives	Committee on Economy, Trade and Industry	METI
House of Councillors	Committee on Economy, Trade and Industry	METI
House of Representatives	Committee on Land, Infrastructure, Transport and Tourism	MLIT
House of Councillors	Committee on Land, Infrastructure, Transport and Tourism	MLIT

Abbreviations: CAO, Cabinet Office; MIC, Ministry of Internal Affairs and Communications; MOJ, Ministry of Justice; MOFA, Ministry of Foreign Affairs of Japan; MOF, Ministry of Finance; MEXT, Ministry of Education, Culture, Sports, Science and Technology; MHLW, Ministry of Health, Labour and Welfare; MAFF, Ministry of Agriculture, Forestry and Fisheries; METI, Ministry of Economy, Trade and Industry; MLIT, Ministry of Land, Infrastructure and Transport.

Figure S2. Per-capita outpatient costs by ministry and year

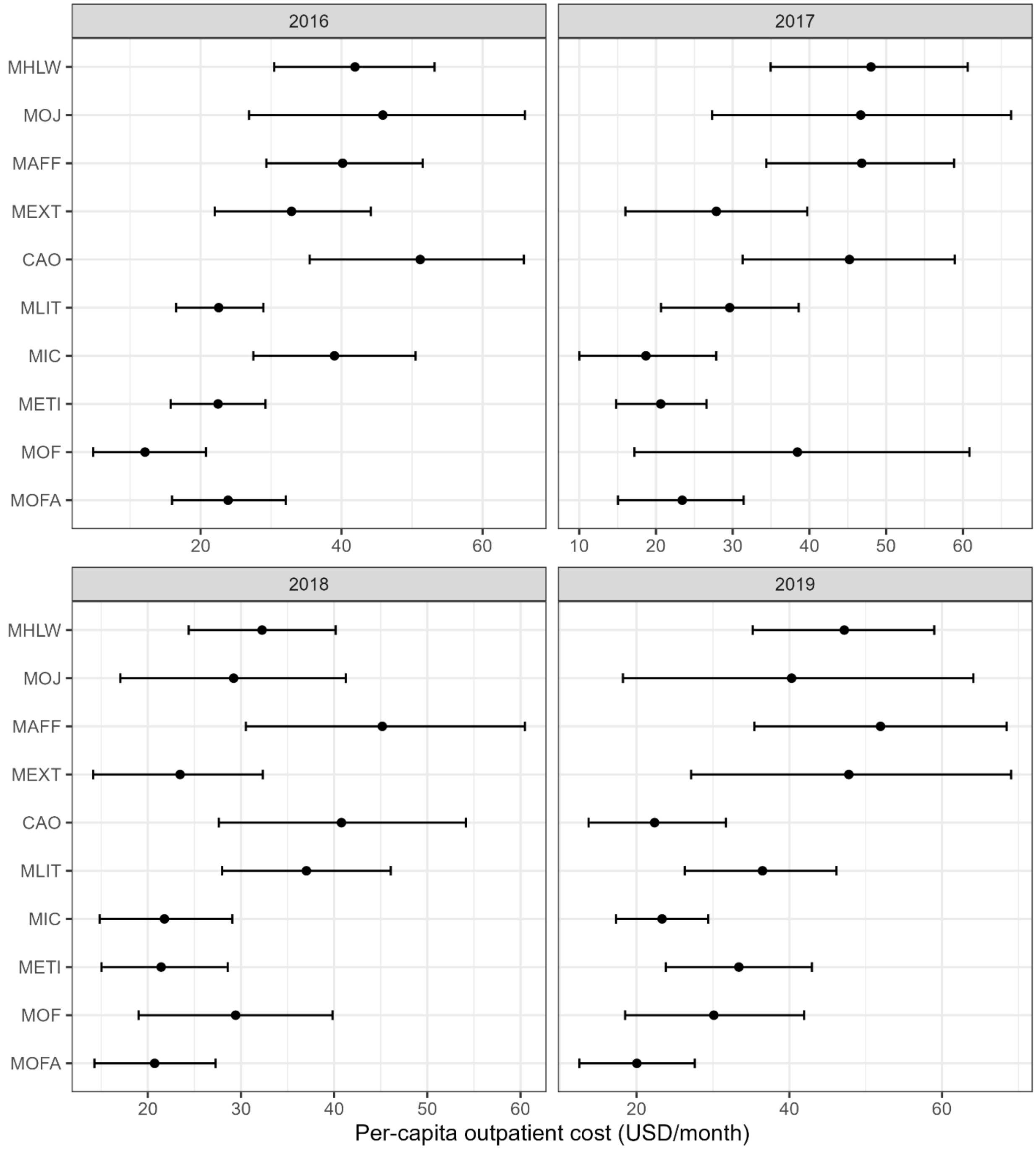

Figure S2. Per-capita outpatient costs by ministry and year (cont.)

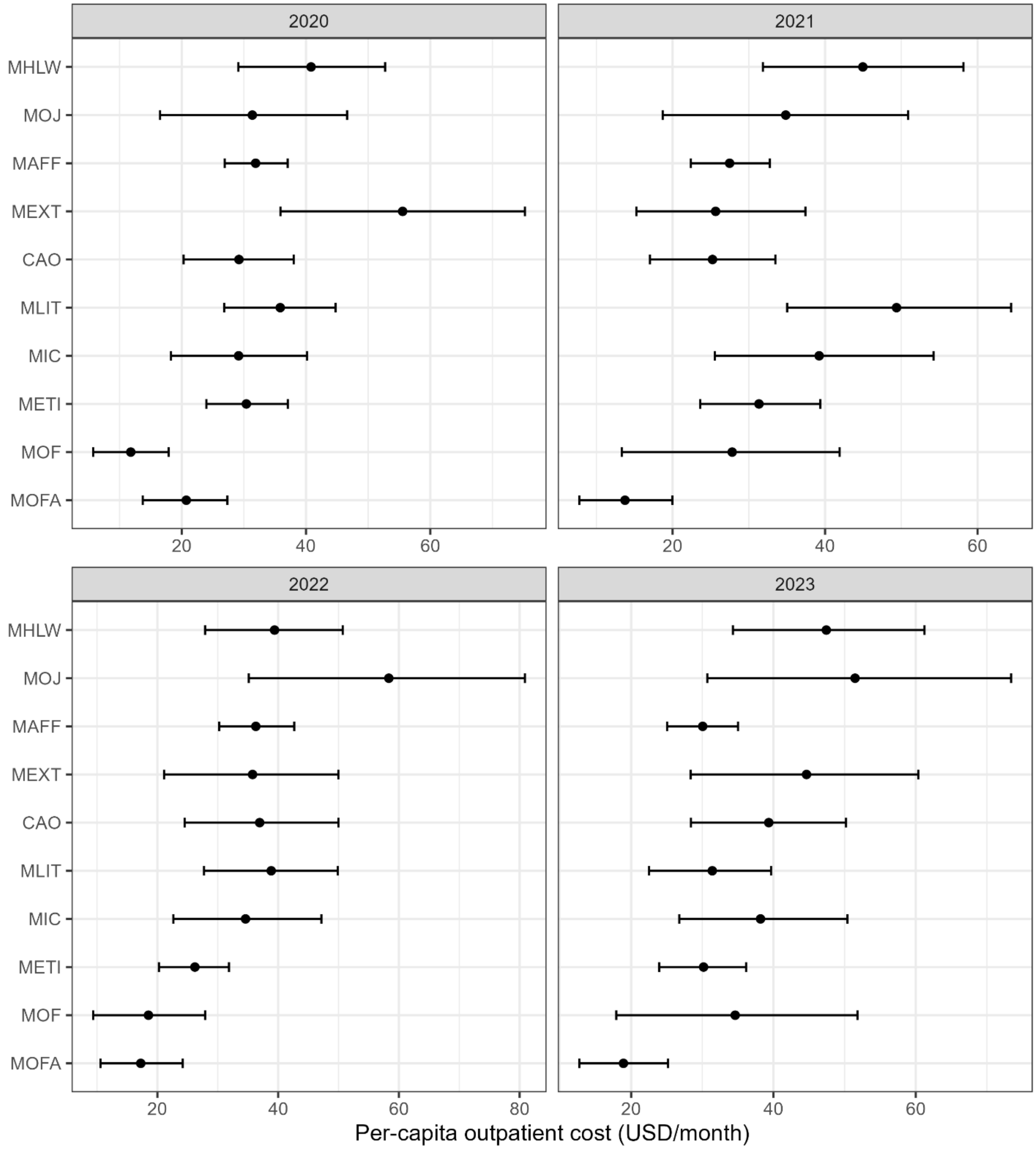

Per-capita outpatient costs are standardized by sex and age groups. Horizontal bars indicate 95% bootstrap confidence intervals.

Abbreviations: CAO, Cabinet Office; MIC, Ministry of Internal Affairs and Communications; MOJ, Ministry of Justice; MOFA, Ministry of Foreign Affairs of Japan; MOF, Ministry of Finance; MEXT, Ministry of Education, Culture, Sports, Science and Technology; MHLW, Ministry of Health, Labour and Welfare; MAFF, Ministry of Agriculture, Forestry and Fisheries; METI, Ministry of Economy, Trade and Industry; MLIT, Ministry of Land, Infrastructure and Transport.

Figure S3. Within-ministry trajectories of deliberation time and outpatient costs

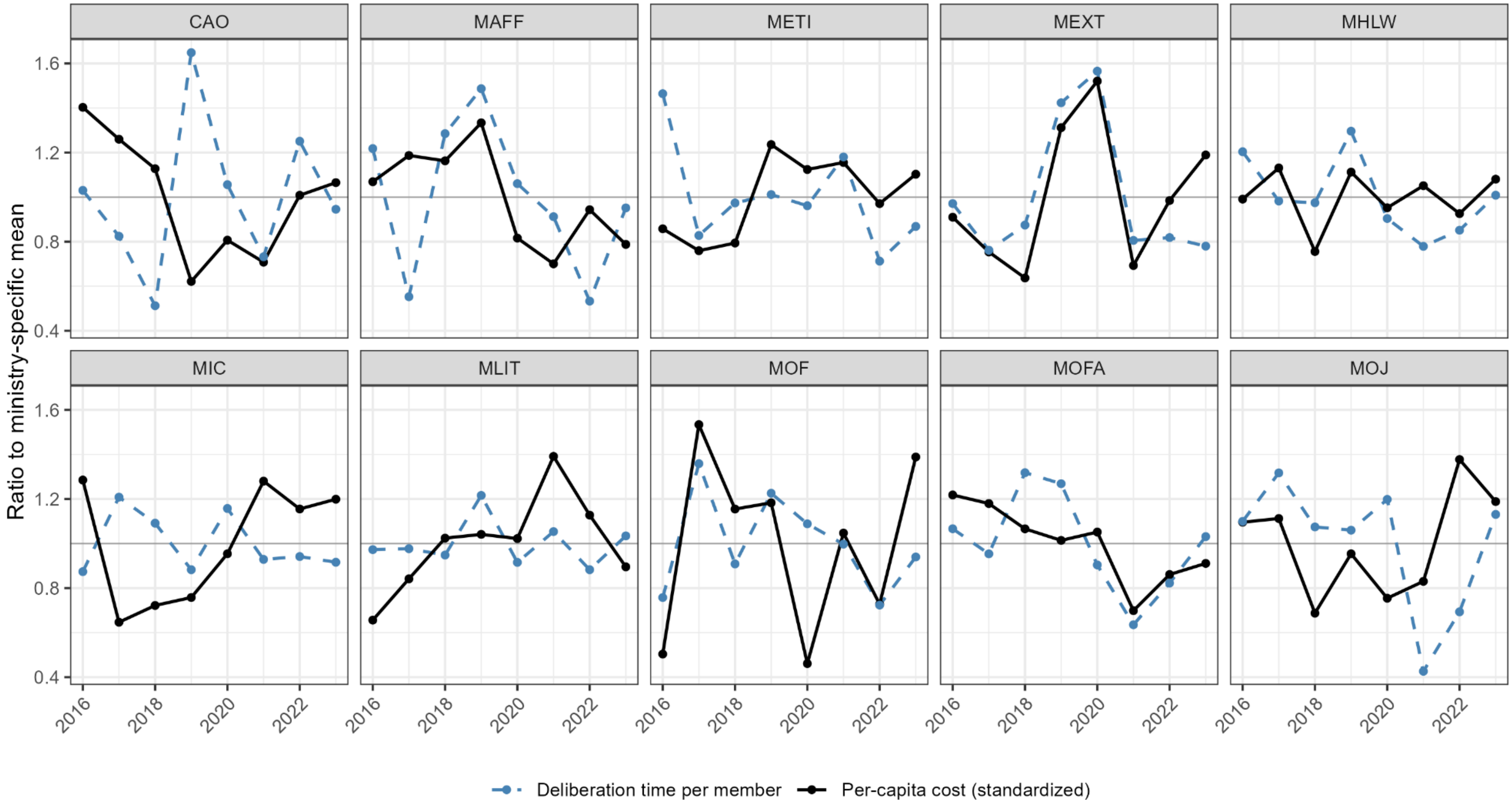

Both series are expressed as the ratio to the ministry-specific mean over the study period (horizontal line at 1); values above 1 indicate years above that ministry's own average. Deliberation time refers to committee deliberation time per insured headquarters member in the previous year; costs are sex- and age-standardized per-capita outpatient costs.

Abbreviations: CAO, Cabinet Office; MIC, Ministry of Internal Affairs and Communications; MOJ, Ministry of Justice; MOFA, Ministry of Foreign Affairs of Japan; MOF, Ministry of Finance; MEXT, Ministry of Education, Culture, Sports, Science and Technology; MHLW, Ministry of Health, Labour and Welfare; MAFF, Ministry of Agriculture, Forestry and Fisheries; METI, Ministry of Economy, Trade and Industry; MLIT, Ministry of Land, Infrastructure and Transport.

Figure S4. Within-ministry association between deliberation time and outpatient costs

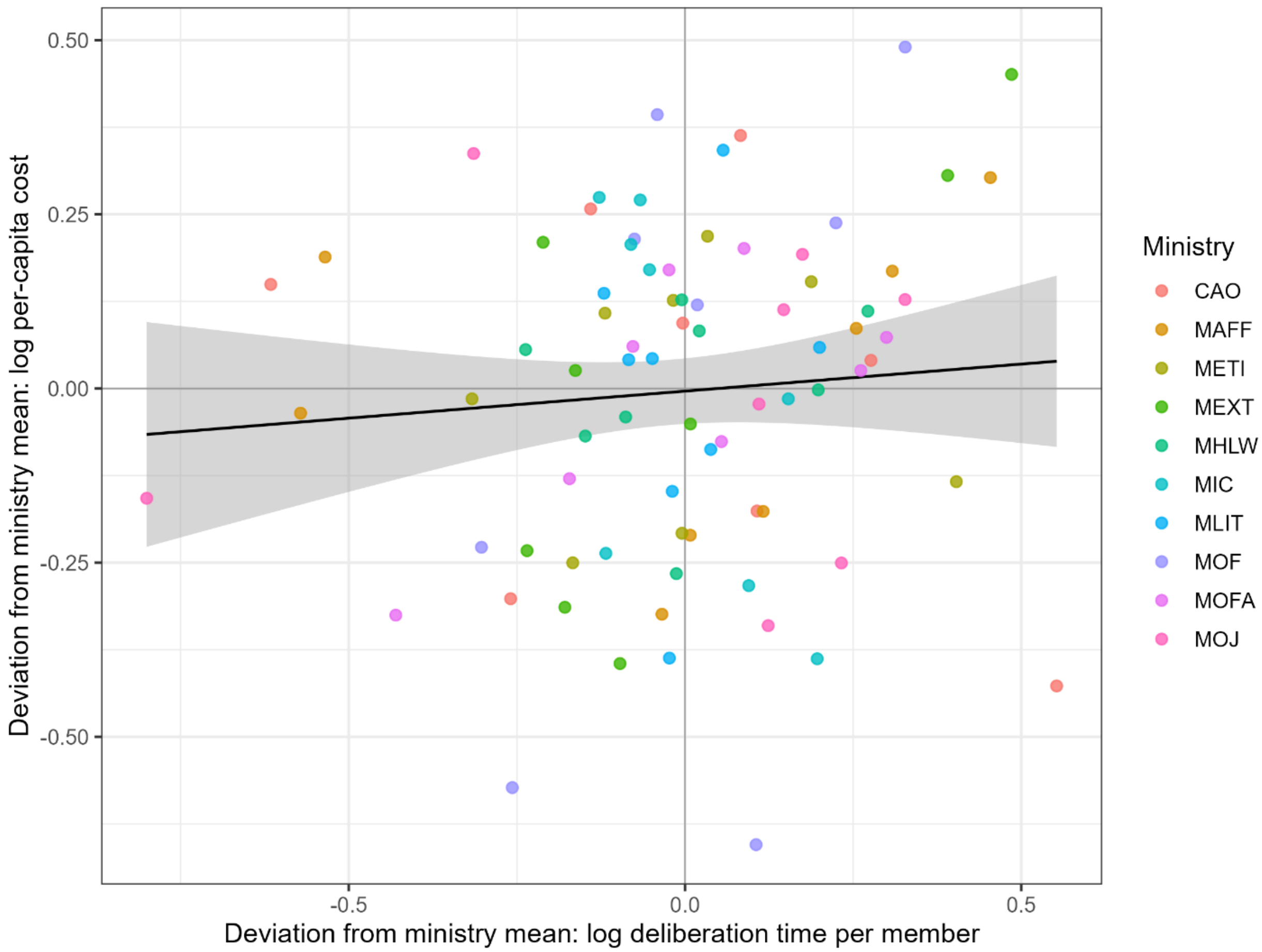

Each point is a ministry-year; both variables are log-transformed and centered on ministry-specific means, so the plot displays only within-ministry variation. The line is a weighted least-squares fit with a 95% confidence band.

Abbreviations: CAO, Cabinet Office; MIC, Ministry of Internal Affairs and Communications; MOJ, Ministry of Justice; MOFA, Ministry of Foreign Affairs of Japan; MOF, Ministry of Finance; MEXT, Ministry of Education, Culture, Sports, Science and Technology; MHLW, Ministry of Health, Labour and Welfare; MAFF, Ministry of Agriculture, Forestry and Fisheries; METI, Ministry of Economy, Trade and Industry; MLIT, Ministry of Land, Infrastructure and Transport.

Figure S5. Estimates in sensitivity analyses

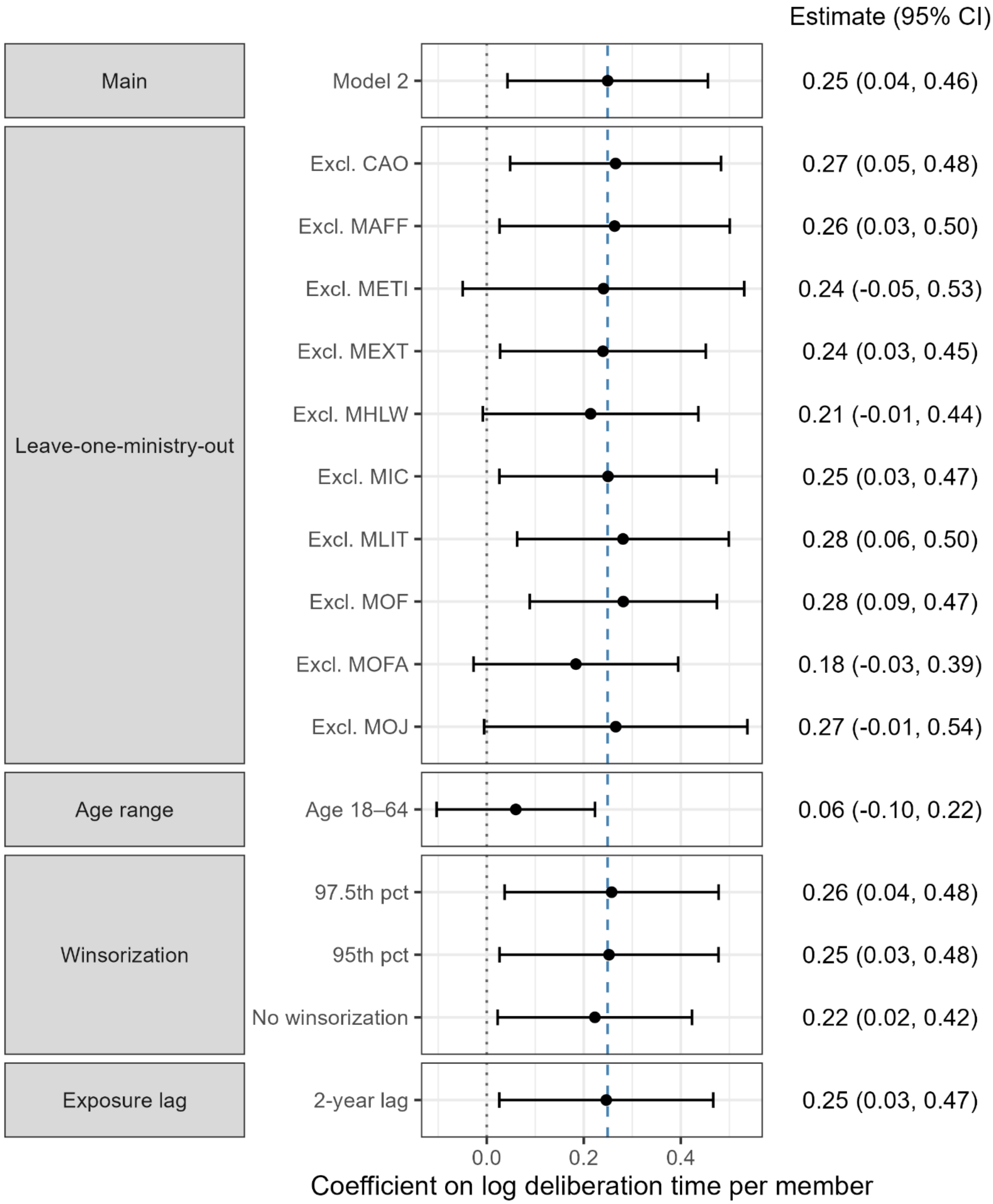

All models were adjusted for year fixed effects. 95% confidence intervals (CI) were estimated using CR2 cluster-robust variance estimation with Satterthwaite degrees of freedom, clustered at the ministry level. The blue dashed line represents the estimate of the main model (Model 2).
Abbreviations: CAO, Cabinet Office; MIC, Ministry of Internal Affairs and Communications; MOJ, Ministry of Justice; MOFA, Ministry of Foreign Affairs of Japan; MOF, Ministry of Finance; MEXT,

Ministry of Education, Culture, Sports, Science and Technology; MHLW, Ministry of Health, Labour and Welfare; MAFF, Ministry of Agriculture, Forestry and Fisheries; METI, Ministry of Economy, Trade and Industry; MLIT, Ministry of Land, Infrastructure and Transport.
